# *Torque teno virus* (TTV) Infection in Patients with Encephalitis

**DOI:** 10.3390/ijms252011177

**Published:** 2024-10-17

**Authors:** Henryk Jurasz, Iwona Bukowska-Ośko, Małgorzata Rydzanicz, Marta Popiel, Tomasz Dzieciątkowski, Karolina Bakuła-Grządka, Marcin Paciorek, Michał Makowiecki, Andrzej Horban, Tomasz Laskus, Marek Radkowski, Karol Perlejewski

**Affiliations:** 1 Department of Immunopathology of Infectious and Parasitic Diseases, Medical University of Warsaw, Pawinskiego 3c, 02-106 Warsaw, Poland; henryk.jurasz@wum.edu.pl (H.J.); iwona.bukowska@wum.edu.pl (I.B.-O.); mszabl@wp.pl (M.P.); mradkowski@wum.edu.pl (M.R.); 2 Department of the Medical Genetics, Medical University of Warsaw, Pawinskiego 3c, 02-106 Warsaw, Poland; malgorzata.rydzanicz@wum.edu.pl; 3 Chair and Department of Medical Microbiology, Medical University of Warsaw, Chałubinskiego 5, 02-004 Warsaw, Poland; dzieciatkowski@wp.pl; 4 Department of Adults Infectious Diseases, Medical University of Warsaw, Wolska 37, 01-201 Warsaw, Poland; mpaciorek@op.pl (M.P.); michalmakowiecki08@gmail.com (M.M.); ahorban@zakazny.pl (A.H.)

**Keywords:** *Torque teno* virus, TTV, encephalitis, neuroinfection

## Abstract

*Torque teno* virus (TTV) is a ssDNA orphan virus belonging to the *Anelloviridae* family, but some recent studies suggested its possible involvement in central nervous system (CNS) pathology. We analyzed serum and cerebrospinal fluid samples (CSF) from 109 patients with encephalitis for TTV infection using serological and molecular testing, virus quantitative measurement, and next-generation sequencing-based (NGS) phylogenetic analysis. TTV noncoding region (UTR) and/or open reading frame 1 (ORF-1) sequences were detected in serum of 86 (79%) patients and in nine (8%) patients in CSF. Five of the latter patients were coinfected with various *entero*- and *herpesviruses*. Anti-TTV-IgG were detected in 80 (73.4%) sera and in two (1.8%) CSF samples, while anti-TTV-IgM were present in three (2.8%) sera and in none of the CSFs. Phylogenic analysis of CSF-derived TTV ORF-1 sequences revealed the presence of three unique variants in one patient. TTV was quantified in five CSF-serum pairs: in two patients viral loads were similar, and in three serum TTV loads were approximately one log higher. Our results suggest at least an occasional replication of TTV in CNS. However, whether TTV could be the cause of encephalitis requires further studies.

## 1. Introduction

*Torque teno* virus (TTV) is a non-enveloped single-stranded circular DNA (3.8 kb) orphan virus closely related to *Torque teno mini* virus (TTMV) and *Torque teno midi* virus (TTMDV) that belongs to the *Anelloviridae* family [1,2]. TTV was isolated in 1997 from three Japanese patients with post-transfusion non-A-E hepatitis; however, further studies excluded TTV as the causative agent of liver injury [2,3,4,5]. Since then, TTV has been tentatively linked to a variety of diseases, including pancreatic [6] and laryngeal cancer [7], diabetes [8], arthritis [9], and several central nervous system (CNS) pathologies, including Alzheimer disease [10] and multiple sclerosis [11]. However, these associations require confirmation in larger studies. Finding any epidemiological association is complicated by the fact that TTV is ubiquitous, and as many as 3.7% to 27.3% of blood donors have specific antibodies [12,13], and up to 65% actively replicate the virus [14].

Encephalitis is caused by infectious agents including bacteria, fungi, and parasites, but mainly by viruses and rarely by autoimmune mechanisms [15]. In the large California Encephalitis Project, viruses were found to be responsible for 69% of cases of encephalitis with infectious etiology [16]. Importantly, in 40% to 80% of patients, the etiology of encephalitis could not be determined, which raises the possibility that some rare or even novel pathogen(s) could play a role in its pathogenesis [16,17,18,19,20,21]. Identification of the causative agent is often essential to determine the appropriate therapy and to predict the outcome [17,22,23]. Two independent studies reported on the presence of TTV-DNA in the CSF of patients with encephalitis, raising the possibility that this infection could play a role in encephalitis pathogenesis, at least occasionally [24,25].

The present study investigated the potential role of TTV in the etiology of encephalitis by employing serological and molecular testing, virus quantification, and next-generation sequencing-based (NGS) phylogenetic analysis. Our study is the largest and most comprehensive analysis of TTV infection in encephalitis patients.

## 2. Results

TTV-DNA was detected in serum of 86 patients (77.98% and 35.78% were positive for UTR and ORF-1, respectively) and in CSF of nine patients (six were positive for UTR and five were positive for ORF-1). All CSF-positive patients were also positive in serum, while only 10.5% of patients who were positive in serum were also positive in CSF. Some clinical, serological, and sequencing data of the nine patients who were TTV-DNA positive in CSF are shown in Table 1.

Anti-TTV-IgG were detected in 80 (73.39%) serum samples and in two (1.83%) CSF samples, while anti-TTV-IgM were found in four (3.67%) sera and in no CSF (Table 2). Among 86 patients who were TTV-DNA positive in serum-specific antibodies, the following were present in 65 (75.58%): 62 were positive for anti-TTV-IgG only; two patients had both anti-TTV-IgM and IgG, and in one only anti-TTV-IgM were detectable.

**Figure 1:**
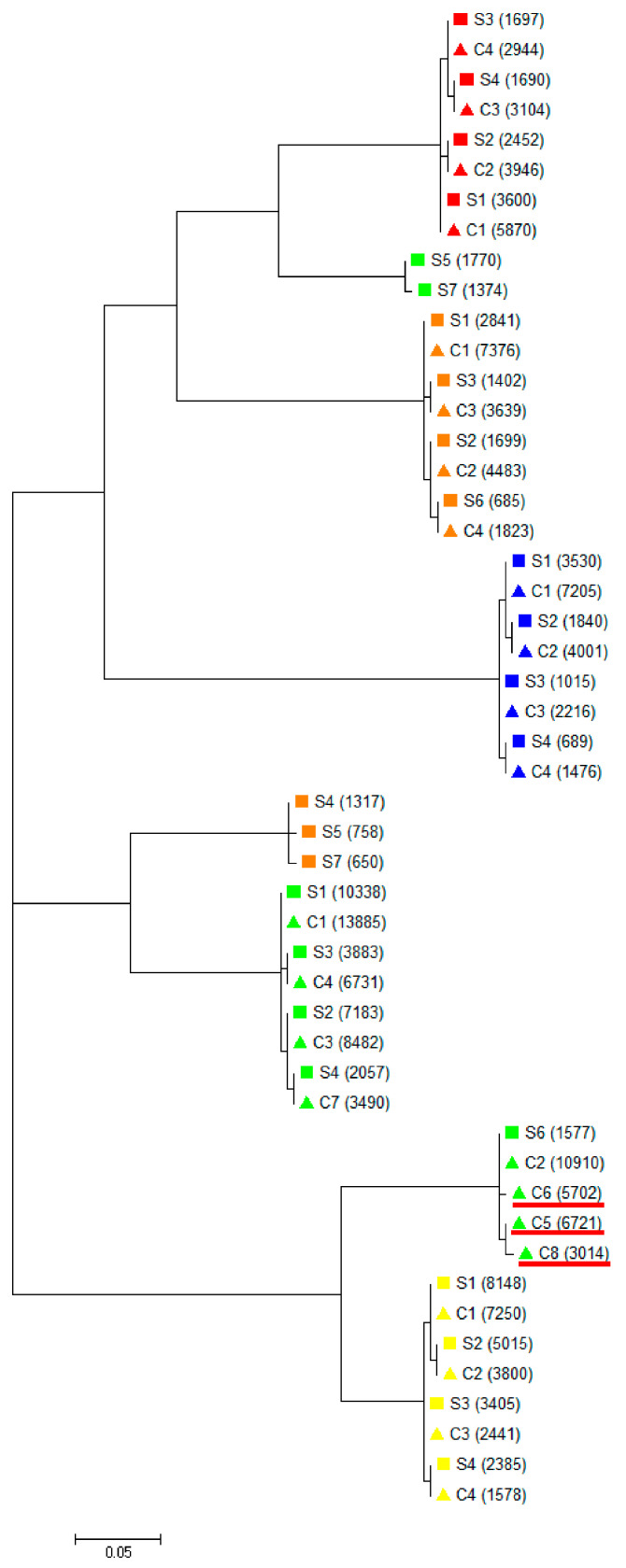
Phylogenetic analysis of the TTV open reading frame 1 (ORF-1) region amplified from serum and CSF in five patients with encephalitis. Phylogenetic trees were generated using MEGA ver. 10. Patient 1 is marked in green, Patient 2 in yellow, Patient 3 in blue, Patient 4 in red, and Patient 5 in orange. Numbers in brackets indicate the number of reads representing each variant. Serum—□S; CSF—ΔC. Unique variants in CSF from patient 5 are underlined in red.

For the purpose of phylogenic analysis, ORF-1 PCR products underwent NGS. Overall, 195,087 raw NGS reads were generated, and after quality check and trimming, the resulting 192,637 reads (an average of 19,509 reads per sample) underwent further analysis. In each of the Patients 2–4, we have found four viral variants that were identically represented in serum and corresponding CSF. In Patient 5, all ORF-1 variants identified in CSF were also present in serum, while the presence of three variants (S4, S5, and S7) was limited to serum. Finally, Patient 1 harbored two unique variants (S5 and S7) in serum and three unique viral variants in CSF; however, all identified nucleotide substitutions in CSF were silent (Figure 1 and Table S1).

TTV viral loads measured in serum samples from Patients 1–3 were 6.3–10 times higher than in the corresponding CSFs, while in Patients 4 and 5 viral loads in serum and CSF were similar, being approximately 350 copies/mL (Table 1).

## 3. Discussion

In the present study, TTV-DNA was detected in the CSF in nine out of 109 (8%) patients with encephalitis. While our study is the largest analysis of TTV infection in patients with encephalitis, several previous pediatric reports already suggested a possible role of this virus in neuroinfections. Ikuta et al. detected TTV in the CSF of a 2-month-old Japanese child with meningitis with the help of NGS-metagenomics [26], while TTMV-G1-3, which is a closely related member of the *Anelloviridae* family, was described for the first time in pediatric patients with acute encephalitis/meningoencephalitis in Ghana [27]. Similarly, another novel TTMV was detected in the CSF of a five-year-old Chinese boy with encephalitis of unknown cause [25].

TTV infection was also reported in other neurological diseases. Maggi et al. analyzed 32 CSF samples collected from patients with various CNS pathologies and found TTV-DNA in three: one patient had non-Hodgkin’s lymphoma, another had progressive multifocal leukoencephalopathy (PML)/AIDS, and the third one suffered from slowly progressive dementia. Interestingly, in the latter patient there were differences between serum- and CSF-derived viral variants [28]. In yet another study, TTV-DNA was identified in CSF in two cases of hydrocephalus, in a case of cranial trauma, and in a patient with trigeminal neuralgia. Surprisingly, patients with cranial trauma harbored TTV subtype 1a in serum and subtype 2c in CSF [29].

Any difference between CSF- and serum-derived TTV variants suggests a possible CNS compartmentalization, so far documented for several other viruses, including the human immunodeficiency virus [30,31], hepatitis C virus [32,33], GBV-C [34], and hepatitis B virus [35,36]. Due to the limited quantity of CSF and/or serum samples, NGS phylogenetic analysis and comparison between compartments could be conducted in only five of our patients. However, one patient harbored three unique CSF-derived variants that were not present in his serum. Although these substitutions were silent, our findings suggest the existence of a separate CNS compartment.

We also conducted quantitative comparisons of TTV loads in CSF and peripheral blood. Three of our patients had viral loads in serum higher by approximately one log than in CSF, while in two patients viral loads in both compartments were similar. Still, this proportion between serum and CSF load is much lower than in the study by Maggi et al., who reported the difference to be as high as 3–4 log10 when analyzing two patients with neurological disorders (a case of non-Hodgkin’s lymphoma and a case of PML/AIDS), and proposed likely blood-brain barrier (BBB) disruption and/or CSF blood contamination as an explanation [28].

Our study is the first to test TTV antibodies in serum and CSF in patients with encephalitis. Typically, TTV-IgM antibodies appear in the blood 10–21 weeks after infection, and their levels decline after approximately 5–11 weeks [13,37] while TTV-IgG become detectable 16 weeks after infection, reaching their maximum titer at five months of virus persistence [37]. However, there are very few studies on the prevalence of TTV antibodies in various clinical conditions and in healthy subjects. Ma et al. found anti-TTV-IgG in 1.2%, 3.7%, 26.8%, and 34.4% of healthy subjects, blood donors, hepatitis A-G, and hepatitis non-A-G patients, respectively [12]. In our study, the prevalence of anti-TTV-IgG was as high as 73%, and this is compatible with the study finding 78% of Polish blood donors to be TTV-DNA positive [38]. Similarly to other studies, we have found that molecular testing based on ORF-1 amplification is less sensitive than the UTR-based PCR approach (36% vs. 78%); [38,39]. Our findings also confirm previous reports finding a good relationship between the presence of anti-TTV-IgG and TTV-DNA in serum [40], since 81.25% of our patients with detectable antibodies were also positive by molecular testing in serum.

In Patient 8, anti-TTV-IgG were detected in CSF but not in serum (the patient was also HSV-1 positive), raising the possibility of intrathecal antibody synthesis in this case. However, only 3% of our patients were TTV-IgM positive in serum, and none was positive in CSF, which suggests that the majority of TTV infections were chronic and thus unlikely to be responsible for the acute bout of encephalitis. It should be emphasized that the mere presence and even replication of TTV in CNS in patients with encephalitis does not imply causality, and among our nine patients with detectable TTV-DNA in CSF, another viral pathogen was identified in five: there were two cases of HSV and single cases of CMV, VZV, and EV infection.

In conclusion, we detected TTV-DNA in CSF in 8% of encephalitis cases, and phylogenetic analysis revealed unique TTV variants in one patient. While our study suggests at least an occasional presence of active replication in the CNS, it is currently unclear whether TTV is the cause of encephalitis or just an ‘innocent bystander’.

## 4. Methods

### 4.1. Patients

Serum and CSF samples were collected from 109 adult patients (≥18 yr; 48 women; 61 men) admitted to the Hospital for Infectious Diseases in Warsaw with a diagnosis of encephalitis between April 2010 and November 2019. Ninety (82%) of these patients were a part of a previously published surveillance study on encephalitis in Poland [20].

Samples were collected in the first 24 h after the patient’s admission and kept frozen at −80 °C until analysis. Encephalitis was diagnosed using the following criteria: (a) presence of altered mental status or consciousness, seizures, or focal neurological signs; (b) CSF test results: white blood cell count ≥ 4 cells/mm^2^ and/or protein level ≥ 0.4 g/L.

CSF samples were tested by PCR/RT-PCR for the presence of human adenovirus and *herpesviruses:* Herpes simplex virus-1 (HSV-1) and Herpes simplex virus-2 (HSV-2), Varicella zoster virus (VZV), Cytomegalovirus (CMV), Human herpesviruses 6 and 7 and *enteroviruses:* Coxsackie A9, A16, B2, B3, B4, B5; ECHO 5, 6, 9, 11, 18, 30 and 71 as described previously [41,42,43,44,45]. In addition, anti-tick-borne encephalitis virus (TBEV) antibodies were tested as part of routine laboratory diagnostics (Serion ELISA classic TBEV IgG and IgM, Institut Virion/Serion GmbH, Würzburg, Germany). Clinical, laboratory, and epidemiological profiles of analyzed patients are presented in Table 2.

### 4.2. TTV ORF-1 and UTR Region Amplification

DNA was extracted from 200 µL of serum and CSF using QIAamp MinElute Virus Spin Kit (QIAGEN, Hilden, Germany) following the manufacturer’s instructions. One nanogram of DNA was subjected to amplification using the FastStart High Fidelity PCR System kit (Roche Diagnostics, Mannheim, Germany). The noncoding region (UTR) was amplified using one-step PCR with primers NG133 and NG147 [46] (143 bp amplicon). Detection of TTV open reading frame 1 region (ORF-1) was based on nested PCR: first round with primers NG059 and NG063, and second round with primers NG061 and NG063 (271 bp amplicon); [46]. Amplification was carried out using an ABI 9700 Thermal Cycler (Applied Biosystems, Waltham, MA, USA). PCR products were visualized using 2% agarose gel electrophoresis. Primer sequences and PCR conditions are shown in Table 3.

### 4.3. Serological Testing

Antibodies against TTV were detected using TTV-IgM and TTV-IgG ELISA Kits (MyBioSource, San Diego, CA, USA) according to the manufacturer’s instructions. Optical density (OD) measurements were performed on a Multiskan FC (Thermo Fisher Scientific, Waltham, MA, USA) photometer.

### 4.4. Next-Generation Sequencing (NGS)

NGS libraries were generated using five nanograms of ORF-1 first-round PCR product and primers compatible with the Illumina MiSeq platform (Illumina, San Diego, CA, USA) [34]. In short, each primer contained: (a) sequences complementary to the adapters on a flow cell; (b) an 8 nt unique index sequence; (c) sequence-specific to Illumina sequencing primers; and (d) sequence-specific primers for the ORF-1. The NGS library was generated using the FastStart High Fidelity PCR System kit (Roche Diagnostics, Mannheim, Germany), and employing the following conditions: initial denaturation at 95 °C for 5 min, followed by 40 cycles of amplification at 95 °C for 30 s, 60 °C for 45 s, 72 °C for 1 min, and final elongation at 72 °C for 7 min. PCR products were visualized on 2% agarose gel and then purified using the QIAquick Gel Extraction Kit (QIAGEN, Germany). Purified and extracted PCR products ranged between 250 and 300 bp in length.

The quality and the average length of a given DNA library were assessed using a bioanalyzer (Agilent Technologies, Santa Clara, CA, USA). Generated libraries were pooled equimolarly and sequenced on Illumina MiSeq (301 base paired-end reads) in two separate runs, according to the manufacturer’s protocol (Illumina, San Diego, CA, USA). Each patient’s serum and CSF sample were sequenced in the same run.

Raw reads were trimmed using cutadapt-1.2.1 [47] and Trimmomantic [48], and all reads under Q25 (phred quality score) were removed using the fastx_artifacts_filter script included in FASTX-Toolkit version 0.13 [49]. Reads of 260 bp were filtered using BBmap version 38.90 [50]. Subsequently, reads were preprocessed (grouping, counting, and frequency arrangement) using FASTX-Toolkit [49]. To reduce the contribution of false positive variants, a cut-off threshold of 2.19, or 2.42%, was applied based on sequencing error specific for each sequencing run. Finally, the remaining sequences were aligned, and phylogenic trees were generated using Clustal X ver.2 [51] and MEGA 11 software [52]. Phylogenetic analysis was performed based on *Torque teno* virus 1 complete genome (GenBank: MH017583.1).

### 4.5. qPCR-Based Quantification of TTV

Non-commercial qPCR was used to compare TTV viral loads between serum and CSF. Two microliters of Fast Start DNA Master SYBR Green I (Roche Diagnostics, Mannheim, Germany), 0.5 μM of TTV-UTR NG133 and NG147 primers, 2 mM MgCl2, and one ng of template DNA were mixed and amplified on the Roche LightCycler II platform (Roche Molecular Biochemicals, Mannheim, Germany) applying the following conditions: 3 min at 95 °C for initial denaturation followed by 45 cycles of 30 s denaturation at 95 °C, 40 s annealing at 60 °C, and 60 s extension at 72 °C. Melting curve analysis was performed to confirm the amplification specificity. Serial dilutions of cloned TTV-UTR amplicon (91–233 nt region of TTV genome) with known template copy number were used as quantitative standards. The qPCR sensitivity was approximately 10^2^ TTV viral copies/mL. All measurements were repeated in an independent run.

## Figures and Tables

**Table 1:** Clinical, laboratory, and NGS data of nine patients with encephalitis who were TTV-DNA-positive in cerebrospinal fluid (CSF).

				CSF Analysis			TTV PCR (ORF1/UTR)		TTV Serology (IgM/IgG)		Number of ORF-1 TTV Changes in NGS (Nucleic/Amino)		TTV Viral Load (Copies/mL)		Other Viral Infection Detected in CSF
Pt.	Sex	Age	Symptoms	Cytosis (/1 µ)	Protein (g/L)	Glucose (mmol/L)	Serum	CSF	Serum	CSF	Serum	CSF	Serum	CSF	
1	M	59	Headache, fever	24	1.42	4.64	+/+	+/+	−/+	−/−	7/7	8/8	2788	435	-
2	F	58	Fever	4	0.23	2.47	+/+	+/+	−/−	−/−	4/4	4/4	2035	321	CMV
3	F	41	Fever, impaired consciousness	9	2.28	2.72	+/+	+/+	−/−	−/−	4/4	4/4	3417	330	-
4	F	59	Fever, movement dysfunctions	4	0.73	2.58	+/+	+/+	−/+	−/−	4/4	4/4	346	370	-
5	F	64	Vomiting, dizziness	13	1.02	2.48	+/+	+/+	−/+	−/−	7/7	4/4	361	358	HSV-1
6	M	59	Impaired consciousness	3	0.81	5.43	−/+	−/+	−/+	−/−	nd **				VZV
7	M	34	Headache, seizures, impaired consciousness	73	1.31	3.4	−/+	−/+	−/+	−/−	nd				EV
8	M	36	Fever, impaired consciousness	1225	1.78	1.26	+/+	−/+	−/−	−/+	nd				HSV-1
9	M	41	Impaired consciousness	70	1.35	2.61	−/+	+/−	−/+	−/−	nd				-

* M, male; F, female; ** nd—not performed (insufficient amount of CSF for NGS analysis and viral load measurements).

**Table 2:** Clinical, laboratory, and epidemiological characteristics of 109 patients with encephalitis.

	All Patients n = 109	TTV-DNA in CSF n = 9 (8.26%)	No TTV-DNA in CSF n = 100 (91.74%)
**Age; median (range)**	42.5 (19–85)	50 (34–64)	42 (19–85)
**Duration of hospital stay; median (range)**	15.85 (2–100)	15 (7–32)	16 (2–100)
**Symptoms and clinical signs; number (%)**			
Fever ≥ 38 °C	49 (44.95)	3 (33.33)	46 (46)
Headache	57 (52.29)	2 (22.22)	55 (55)
Alerted mental status	20 (18.35)	1 (11.11)	19 (19)
Seizures	16 (14.68)	1 (11.11)	15 (15)
Vomiting	4 (3.67)	1 (11.11)	3 (3)
**CSF analysis; median (range)**			
Cytosis	76 (1–2153)	177 (3–1225)	102 (1–2153)
Lymphocytes %	78 (12–100)	71 (43–87)	78 (12–100)
Protein level g/L	0.76 (0.11–3.33)	1.265 (0.23–2.28)	0.72 (0.11–3.33)
Glucose level mmol/L	3.65 (1.26–7.8)	2.77 (1.26–4.64)	3.72 (2.17–7.80)
**Encephalitis etiology; number (%)**			
TBEV	6 (6.42)	0	6 (6)
HSV	21 (22.02)	2 (22)	19 (19)
CMV	2 (2.75)	1 (11)	1 (1)
VZV	5 (4.59)	1 (11)	4 (4)
EV	7 (6.42)	1 (11)	6 (6)
Unknown etiology	68 (62.39)	4 (44)	64 (64)
**TTV IgG/IgM; number (%)**			
Serum	80 (73.39)/4 (3.67)	6 (66.67)/0	74 (74.0)/4 (4)
CSF	2 (1.83)/0	1 (11.11)/0	1 (1)/0
**TTV DNA in serum; number (%)**			
ORF-1	39 (35.78)	6 (66.67)	33 (33)
UTR	85 (77.98)	9 (100)	76 (76)
ORF-1 or UTR	86 (78.89)	9 (100)	77 (77)

**Table 3:** Primers and PCR conditions used for amplification of TTV noncoding (UTR) and open reading frame 1 (ORF-1) region [46]. Nucleotide numbering followed the MH017583.1 (GenBank) sequence.

TTV Region	Round of PCR	Primers (TTV Genome Position)	Amplicon (bp)	PCR Conditions	
**UTR region**	I PCR	Forward: 5′-GTA AGT GCA CTT CCG AAT GGC TGA G-3′ (91–115) Reverse: 5′-GCC AGT CCC GAG CCC GAA TTG CC-3 (211–233)	143	95 °C, 3 min	
				95 °C, 30 s	35 cycles
				60 °C, 40 s	
				72 °C, 60 s	
				72 °C, 7 min	
**ORF-1 region**	I PCR	Forward: 5′-ACA GAC AGA GGA GAA GGC AAC ATG-3′ (1900–1923) Reverse: 5′-CTG GCA TTT TAC CAT TTC CAA AGT T-3′ (2161–2185)	286	95 °C, 3 min	
				95 °C, 30 s	35 cycles
				60 °C, 45 s	
				72 °C, 60 s	
				72 °C, 7 min	
	II PCR	Forward: 5′-GGC AAC ATG YTR TGG ATA GAC TGG-3′ (1915–1938) Reverse: 5′-CTG GCA TTT TAC CAT TTC CAA AGT T-3′ (2161–2185)	271	95 °C, 3 min	
				95 °C, 30 s	20 cycles
				60 °C, 45 s	
				72 °C, 60 s	
				72 °C, 7 min	

## Data Availability

The datasets generated during and/or analyzed during the current study are available from the corresponding author on reasonable request.

## Supplementary Materials

The following supporting information can be downloaded at: https://www.mdpi.com/article/10.3390/ijms252011177/s1.
