# Supplementary material for: Torque teno virus (TTV) Infection in Patients with Encephalitis
Source: Int J Mol Sci. 2024 Oct 17;25(20):11177. doi: 10.3390/ijms252011177 (PMC11508335; doi:10.3390/ijms252011177)
Supplement: Supplementary file 1 [file ijms-25-11177-s001.zip › ijms-3232986-supplementary.pdf]

**Table S1.** Comparison of nucleic (**A**) and amino acid (**B**) sequences of TTV ORF-1 region amplified from serum and CSF from five patients with confirmed TTV-DNA in CNS. Numbers in brackets represent the number of reads representing each variant. Shading indicates sequences unique to CSF.

**A)**

**Patient 1**

```
S1(10338) AACATGTTGTGGATAGACTGGATATCAAAGACAGACTCAATCTATGACAAAGCACGCAGCAAATGTTTAATAGAAAAATTACCCATGTGGGCCGCGGTATATGGCTACGCAGAATACTGTGCCAAAAGCA
S2(7183)  ........A.........................................................................................................................
S3(3883)  ......C...........................................................................................................................
S4(2057)  ......C.A.........................................................................................................................
S5(1770)  .....................C..A.C..A.AT......CAA...TCA...A...AG.....G...C.C.....G..C..G..TC.C........A.....C..A...A..........CAG....GTA.
S6(1577)  .....................C..AGC..A.A.A.TATG.A...........T..AA..T.....C......TC.G.CC....TC.A.....A..A.C......A..TTT.....TT.....A.....T.
S7(1374)  ......C..............C..A.C..A.AT......CAA...TCA...A...AG.....G...C.C.....G..C..G..TC.C........A.....C..A...A..........CAG....GTA.
C1(13885) ..................................................................................................................................
C2(10910) .....................C..AGC..A.A.A.TATG.A...........T..AA..T.....C......TC.G.CC....TC.A.....A..A.C......A..TTT.....TT.....A.....T.
C3(8482)  ........A.........................................................................................................................
C4(6731)  ......C...........................................................................................................................
C5(6721)  ........A............C..AGC..A.A.A.TATG.A...........T..AA..T.....C......TC.G.CC....TC.A.....A..A.C......A..TTT.....TT.....A.....T.
C6(5702)  ......C..............C..AGC..A.A.A.TATG.A...........T..AA..T.....C......TC.G.CC....TC.A.....A..A.C......A..TTT.....TT.....A.....T.
C7(3490)  ......C.A.........................................................................................................................
C8(3014)  ......C.A............C..AGC..A.A.A.TATG.A...........T..AA..T.....C......TC.G.CC....TC.A.....A..A.C......A..TTT.....TT.....A.....T.

S1(10338) CAGGAGACTCTAACATAGACATGAACGCCAGAGTAGTTATGAGATGTCCATACACTGTTCCCCAAATGATAGACACAAGCAATCCCCTCAGGGGCTTTATACCGTACAGCTTTAACTTTGGAAATGG
S2(7183)  ...............................................................................................................................
S3(3883)  ...............................................................................................................................
S4(2057)  ...............................................................................................................................
S5(1770)  ..........A........ACAC...TG....TGT.........A.C..C.....A..A..A...C.CT.....CAC.A...........CA..A.A.G....C.......................
S6(1577)  ........CAA..T...C.T.....T......C..C.A..A...A....C.TT..A.AC......C.AC...TACAC.CAG.C...ACA.AA......G.T..C...TCT..A..............
S7(1374)  ..........A........ACAC...TG....TGT.........A.C..C.....A..A..A...C.CT.....CAC.A...........CA..A.A.G....C.......................
C1(13885) ...............................................................................................................................
C2(10910) ........CAA..T...C.T.....T......C..C.A..A...A....C.TT..A.AC......C.AC...TACAC.CAG.C...ACA.AA......G.T..C...TCT..A..............
C3(8482)  ...............................................................................................................................
C4(6731)  ...............................................................................................................................
C5(6721)  ........CAA..T...C.T.....T......C..C.A..A...A....C.TT..A.AC......C.AC...TACAC.CAG.C...ACA.AA......G.T..C...TCT..A..............
C6(5702)  ........CAA..T...C.T.....T......C..C.A..A...A....C.TT..A.AC......C.AC...TACAC.CAG.C...ACA.AA......G.T..C...TCT..A..............
C7(3490)  ...............................................................................................................................
C8(3014)  ........CAA..T...C.T.....T......C..C.A..A...A....C.TT..A.AC......C.AC...TACAC.CAG.C...ACA.AA......G.T..C...TCT..A..............
```

**Patient 2**

```
S1(8148)  AACATGTTATGGATAGACTGGCTTACTAAAAAAAACATGGAATATGACAAAGTGCAAAGCAAGTGCCTAGTAGCAGACCTACCACTGTGGGCAGCAGCATATGGGTATTTAGAATTCTGCTCTAAAAGCA
S2(5015)  ......C...........................................................................................................................
S3(3405)  ........G.........................................................................................................................
S4(2385)  ......C.G.........................................................................................................................
C1(7250)  ..................................................................................................................................
C2(3800)  ......C...........................................................................................................................
C3(2441)  ........G.........................................................................................................................
C4(1578)  ......C.G.........................................................................................................................

S1(8148)  CAGGAGACACAAACATACACATGAATGCCAGACTACTAATAAGAAGTCCCTTTACAGACCCCCAGCTAATAGTACACACAAACCCCAATAAAGGCTTTGTACCTTATTCTTTAAACTTTGGAAATGG
S2(5015)  ...............................................................................................................................
S3(3405)  ...............................................................................................................................
S4(2385)  ...............................................................................................................................
C1(7250)  ...............................................................................................................................
C2(3800)  ...............................................................................................................................
C3(2441)  ...............................................................................................................................
C4(1578)  ...............................................................................................................................
```

**Patient 3**

```
S1(3530)  AACATGTTATGGATAGACTGGCTAAGCAAACCAGACAGCATATACGACCCCTCTAAGAGCAAATGCCTACTAAAAGACTTTCCCCTATGGTGCATGGTATACGGGTACGCAGACTACTGCAGAAAAGTCA
S2(1840)  ......C...........................................................................................................................
S3(1015)  ........G.........................................................................................................................
S4(689)   ......C.G.........................................................................................................................
C1(7205)  ..................................................................................................................................
C2(4001)  ......C...........................................................................................................................
C3(2216)  ........G.........................................................................................................................
C4(1476)  ......C.G.........................................................................................................................

S1(3530)  CAGGAGACTCAGCCATACTACTAGACAGCAGAGTAGTAATAAGATGCCCGTACACATACCCTCAACTTATAAAACACAACAATGACAACTGGGGATTCGTCCCCTACAGCGAAAACTTTGGAAATGG
S2(1840)  ...............................................................................................................................
S3(1015)  ...............................................................................................................................
S4(689)   ...............................................................................................................................
C1(7205)  ...............................................................................................................................
C2(4001)  ...............................................................................................................................
C3(2216)  ...............................................................................................................................
C4(1476)  ...............................................................................................................................
```

**Patient 4**

```
S1(3600)  AACATGTTGTGGATAGACTGGCTAACTAAAGATACCTCAGTATATGACAAAGCACAGAGTAAATGTCTGATACAAGACATGCCCTTGTGGGCCTCTGTGTACGGATTCTCCGAGTACTGCAGTAAAGTAA
S2(2452)  ......C...........................................................................................................................
S3(1697)  ........A.........................................................................................................................
S4(1690)  ......C.A.........................................................................................................................
C1(5870)  ..................................................................................................................................
C2(3946)  ......C...........................................................................................................................
C3(3104)  ......C.A.........................................................................................................................
C4(2944)  ........A.........................................................................................................................

S1(3600)  CAGGAGACACAAACATAGAACACAACTGCAGATGTGTTATTAGAAGCCCCTACACAGTACCACAACTGTTAGATCACAACAACCCCCTCAGGGGATACGTGCCCTACAGTTTTAACTTTGGAAATGG
S2(2452)  ...............................................................................................................................
S3(1697)  ...............................................................................................................................
S4(1690)  ...............................................................................................................................
C1(5870)  ...............................................................................................................................
C2(3946)  ...............................................................................................................................
C3(3104)  ...............................................................................................................................
C4(2944)  ...............................................................................................................................
```

**Patient 5**

```
S1(2841)  AACATGTTGTGGATAGACTGGGTATCTAAATATGACTCTGTATACTCAAAGACACAGAGCAAATGCTTAATAGAAGGCCTACCTCTGTGGGCCGCAGTTTATGGATATGCAGAATACTGCAGCAAAGCCA
S2(1699)  ........A.........................................................................................................................
S3(1402)  ......C...........................................................................................................................
S4(1317)  .....................C....A...ACA.....CA....TGAC.....C.GC.........C.T......AAG.....C..A.....AT....A..C..G..C...........TGC...GAG..
S5(758)   ........A............C....A...ACA.....CA....TGAC.....C.GC.........C.T......AAG.....C..A.....AT....A..C..G..C...........TGC...GAG..
S6(685)   ......C.A.........................................................................................................................
S7(650)   ......C..............C....A...ACA.....CA....TGAC.....C.GC.........C.T......AAG.....C..A.....AT....A..C..G..C...........TGC...GAG..
C1(7376)  ..................................................................................................................................
C2(4483)  ........A.........................................................................................................................
C3(3639)  ......C...........................................................................................................................
C4(1823)  ......C.A.........................................................................................................................
```

```
S1(2841)   CAGGAGACACAAACATAGAACAAAACTGCAGAGTAGTTATTAGAAGCCCCTTCACTAACCCTCAGCTACTAGACCATAACAACCCTCTAAGAGGGTACGTCCCCTACTCAATAAACTTTGGAAATGG
S2(1699)   ...............................................................................................................................
S3(1402)   ...............................................................................................................................
S4(1317)   .C......T..........CATG...GC.........C..A..GT......A...C.C...C..AA.GA.....ACC......GAC..G..G..C.T.A.AGTA...AGCT.T..............
S5(758)    .C......T..........CATG...GC.........C..A..GT......A...C.C...C..AA.GA.....ACC......GAC..G..G..C.T.A.AGTA...AGCT.T..............
S6(685)    ...............................................................................................................................
S7(650)    .C......T..........CATG...GC.........C..A..GT......A...C.C...C..AA.GA.....ACC......GAC..G..G..C.T.A.AGTA...AGCT.T..............
C1(7376)   ...............................................................................................................................
C2(4483)   ...............................................................................................................................
C3(3639)   ...............................................................................................................................
C4(1823)   ...............................................................................................................................
```

**B)**

**Patient 1**

```
S1(10338) NMLWIDWISKTDSIYDKARSKCLIEKLPMWAAVYGYAEYCAKSTGDSNIDMNARVVMRCPYTVPQMIDTSNPLRGFIPYSFNFGN
S2(7183)  .....................................................................................
S3(3883)  .....................................................................................
S4(2057)  .....................................................................................
S5(1770)  .......LT.N..Q.S.TQ......N..L.......T...S.V......EH.C.C...S......LL.HN...T.YV........
S6(1577)  .......L..KNMN...VQ.....SD..L...A...L.F.......Q..H....LLI.S.F.D..LLVHTD.TK..V...L....
S7(1374)  .......LT.N..Q.S.TQ......N..L.......T...S.V......EH.C.C...S......LL.HN...T.YV........
C1(13885) .....................................................................................
C2(10910) .......L..KNMN...VQ.....SD..L...A...L.F.......Q..H....LLI.S.F.D..LLVHTD.TK..V...L....
C3(8482)  .....................................................................................
C4(6731)  .....................................................................................
C5(6721)  .......L..KNMN...VQ.....SD..L...A...L.F.......Q..H....LLI.S.F.D..LLVHTD.TK..V...L....
C6(5702)  .......L..KNMN...VQ.....SD..L...A...L.F.......Q..H....LLI.S.F.D..LLVHTD.TK..V...L....
C7(3490)  .....................................................................................
C8(3014)  .......L..KNMN...VQ.....SD..L...A...L.F.......Q..H....LLI.S.F.D..LLVHTD.TK..V...L....
```

**Patient 2**

```
S1(8148)  NMLWIDWLTKKNMEYDKVQSKCLVADLPLWAAAYGYLEFCSKSTGDTNIHMNARLLIRSPFTDPQLIVHTNPNKGFVPYSLNFGN
S2(5015)  .....................................................................................
S3(3405)  .....................................................................................
S4(2385)  .....................................................................................
C1(7250)  .....................................................................................
C2(3800)  .....................................................................................
C3(2441)  .....................................................................................
C4(1578)  .....................................................................................
```

**Patient 3**

```
S1(3530)  NMLWIDWLSKPDSIYDPSKSKCLLKDFPLWCMVYGYADYCRKVTGDSAILLDSRVVIRCPYTYPQLIKHNNDNWGFVPYSENFGN
S2(1840)  .....................................................................................
S3(1015)  .....................................................................................
S4(689)   .....................................................................................
C1(7205)  .....................................................................................
C2(4001)  .....................................................................................
C3(2216)  .....................................................................................
C4(1476)  .....................................................................................
```

**Patient 4**

```
S1(3600)  NMLWIDWLTKDTSVYDKAQSKCLIQDMPLWASVYGFSEYCSKVTGDTNIEHNCRCVIRSPYTVPQLLDHNNPLRGYVPYSFNFGN
S2(2452)  .....................................................................................
S3(1697)  .....................................................................................
S4(1690)  .....................................................................................
C1(5870)  .....................................................................................
C2(3946)  .....................................................................................
C3(3104)  .....................................................................................
C4(2944)  .....................................................................................
```

**Patient 5**

```
S1(2841)  NMLWIDWVSKYDSVYSKTQSKCLIEGLPLWAAVYGYAEYCSKATGDTNIEQNCRVVIRSPFTNPQLLDHNNPLRGYVPYSINFGN
S2(1699)  .....................................................................................
S3(1402)  .....................................................................................
S4(1317)  .......L..T..I.D..R......K.....S........A.S...S..DM.A.....C.Y.T..MI.T..D...FIV..F....
S5(758)   .......L..T..I.D..R......K.....S........A.S...S..DM.A.....C.Y.T..MI.T..D...FIV..F....
S6(685)   .....................................................................................
S7(650)   .......L..T..I.D..R......K.....S........A.S...S..DM.A.....C.Y.T..MI.T..D...FIV..F....
C1(7376)  .....................................................................................
C2(4483)  .....................................................................................
C3(3639)  .....................................................................................
C4(1823)  .....................................................................................
```
